# Covalently anchoring silver nanoclusters Ag_44_ on modified UiO-66-NH_2_ with Bi_2_S_3_ nanorods and MoS_2_ nanoparticles for exceptional solar wastewater treatment activity

**DOI:** 10.1038/s41598-023-44819-8

**Published:** 2023-10-17

**Authors:** Mostafa Farrag

**Affiliations:** Chemistry Department, Faculty of Science, Assiut University, Assiut, 71516 Egypt

**Keywords:** Chemistry, Catalysis, Environmental chemistry, Green chemistry, Photochemistry, Physical chemistry, Surface chemistry

## Abstract

For the first time, covalently anchoring size selected silver nanoclusters [Ag_44_(MNBA)_30_] on the Bi_2_S_3_@UiO-66-NH_2_ and MoS_2_@UiO-66-NH_2_ heterojunctions were constructed as novel photocatalysts for photodegradation of methylene blue (MB) dye. The anchoring of Ag_44_ on MoS_2_@UiO-66-NH_2_ and Bi_2_S_3_@UiO-66-NH_2_ heterojunctions extended the light absorption of UiO-66-NH_2_ to the visible region and improved the transfer and separation of photogenerated charge carriers through the heterojunctions with a unique band gap structure. The UV–Vis-NIR diffuse reflectance spectroscopic analysis confirmed that the optical absorption properties of the UiO-66-NH_2_ were shifted from the UV region at 379 nm to the visible region at ~ 705 nm after its doping with Bi_2_S_3_ nanorods and Ag_44_ nanoclusters (Bi_2_S_3_@UiO-66-NH-S-Ag_44_). The prepared Bi_2_S_3_@UiO-66-NH-S-Ag_44_ and MoS_2_@UiO-66-NH-S-Ag_44_ photocatalysts exhibited exceptional photocatalytic activity for visible light degradation of MB dye. The photocatalysts exhibited complete decolorization of the MB solution (50 ppm) within 90 and 120 min stirring under visible light irradiation, respectively. The supper photocatalytic performance and recycling efficiency of the prepared photocatalysts attributed to the covalent anchoring of the ultra-small silver clusters (Ag_44_) on the heterojunctions surface. The X-ray photoelectron spectroscopic analysis confirmed the charge of the silver clusters is zero. The disappearance of the N–H bending vibration peak of primary amines in the FTIR analysis of Bi_2_S_3_@UiO-66-NH-S-Ag_44_ confirmed the covalent anchoring of the protected silver nanoclusters on the UiO-66-NH_2_ surface via the condensation reaction. The Bi_2_S_3_@UiO-66-NH-S-Ag_44_ catalyst exhibited excellent recyclability efficiency more than five cycles without significant loss in activity, indicating their good potential for industrial applications. The texture properties, crystallinity, phase composition, particle size, and structural morphology of the prepared photocatalysts were investigated using adsorption–desorption N_2_ isotherms, X-ray diffraction (XRD), HR-TEM, and FE-SEM, respectively.

## Introduction

Due to population growth and heavy consumption of natural resources, freshwater is becoming increasingly polluted by heavy metal ions and organic compounds^1,2^. More than 100,000 toxic and nontoxic dyes are being used for industrial purposes, a significant amount of which is released into water resources after processing^3,4^. Solar photodegradation is the best technique for the removal of dye contaminates from water^5–7^.

In our previous work, a TiO_2_ nanostructures were modified with two different metal chalcogenides (CuS and MoS_2_), that showed high efficiency (98%) in the photodegradation of methylene blue dye under UV–Vis light irradiation^8^. Monodispersed bare silver nanoclusters with an average particle size of 1.2 nm were synthesized without protecting ligand and deposited inside the pores of a titanium dioxide modified mesoporous MCM-41 utilizing the novel strong electrostatic adsorption (SEA) technique^6^. The performance of the synthesized photocatalysts was tested by photocatalytic degradation of MB dye under visible light irradiation^6^.

Recently, metal chalcogenides semiconductor-based photocatalysts, such as ZnS, CdS, CuS, and MoS_2_ have attracted considerable attention due to their efficient photocatalytic activity toward the degradation of organic pollutants, CO_2_ reduction, and water splitting, because of their low cost, narrow band gaps, relative safety, thermal stability and environmental friendly^9–12^. However, the metal chalcogenides suffer from their lower surface area, therefore constructing heterojunctions (chalcogenides/Metal–organic frameworks) photocatalysts have been considered to be an effective method to enhance the photocatalytic performance of the metal chalcogenides. A series of heterojunctions structures were prepared, such as CdS@NH_2_-MIL-125(Ti), Bi_2_S_3_@ZiF-8(Zn), Ag_3_PO_4_@UiO-66, and In_2_S_3_@UiO-66 that exhibited highly photodegradation efficiencies for removal several organic dyes and pollutants such as rhodamine (RhB), methyl orange (MO), and phenol and oxytetracycline (OTC)^13–16^. Most importantly, as a supporting matrix, the heterojunctions can efficiently disperse semiconductor photocatalysts and provide additional channels for the timely separation of photoexcited charge carriers^17^.

Metal–organic frameworks (MOFs), which are made up of metal ions linked together with organic linkers have been recognized as ideal materials due to their large surface area and tunable structures^18,19^. UiO-66 (Universitetet i Oslo) is an archetypal MOFs that is built up from [Zr_6_O_4_(OH)_4_(CO_2_)_12_] clusters linked with terephthalic acid^20,21^. The structure framework includes octahedral and tetrahedral cages in a 1:2 ratio, suitable for loading metal precursors^20,21^. UiO-66 and NH_2_-UiO-66 were used in many applications due to their resistance toward a variety of organic solvents and high thermal and chemical stability^18^, as well as the acidic sites of NH_2_-UiO-66 that come from the Lewis acidity of the unsaturated Zr metal sites that play a significant role in the hydrolysis of NaBH_4_^18^.

Loading MOFs with metal chalcogenide semiconductors makes them more promising for absorbing light^17,22–24^, whereas pure MOFs have limited spectral absorption. One of the most effective ways to improve the photocatalytic efficiency of MOFs is through heterojunction construction, which can limit charge recombination and increase light absorption in the visible light region^17,22^. Bismuth sulfide (Bi_2_S_3_) is a typical lamellar-structure semiconductor with a narrow band gap (~ 1.3 eV)^22^, however, its application is limited due to its easy photocorrosion. As a result, combining Bi_2_S_3_ with MOFs to form heterojunction structures may be an option for overcoming the shortcomings of the two materials^17,22^.

To further enhance the photocatalytic performance of the MOFs, metal nanoparticles have been used as doped materials not only to retard the electron–hole recombination but also to enhance visible light absorption^18,19, 24–33^. Recently, protected metal nanoclusters have been used in many applications, because of their size-dependent optical and electronic properties, which differ significantly from both the single atom and bulk properties. In our previous work, we prepared several monodispersed gold^34–36^, silver^6,7, 37, 38^, platinum^5,39^, and palladium^5,40, 41^ nanoclusters.

In this work, the NH_2_-UiO-66 was chosen as a supporting matrix for many reasons such as ultrahigh porosity, high thermal and chemical stability, and high surface area^18,19^. Moreover, the negatively charged (–NH_2_) surface of NH_2_-UiO-66 allows metal ions to tightly anchor and wrap and facilitate the rapid fabrication of the Bi_2_S_3_@UiO-66-NH_2_ and MoS_2_@UiO-66-NH_2_ heterojunctions. To improve the performance of the heterojunctions in the solar photodegradation of MB dye, the size selected silver nanoclusters [Ag_44_(MNBA)_30_] is anchored onto the Bi_2_S_3_@UiO-66-NH_2_ and MoS_2_@UiO-66-NH_2_ surface through a covalent interfacial reaction (condensation reaction) between the amine groups of UiO-66-NH_2_ and the carboxylic groups of the protecting agent (5-mercapto-2-nitrobenzoic acid). For the first time, size selected silver nanoclusters were used as doped metal nanoparticles over the prepared heterojunctions. The field emission scanning electron microscopy (FE-SEM) results confirms the morphology of Bi_2_S_3_ and MoS_2_ are nanorods and nanoparticles, respectively. Moreover, they are interspersed on the surface of NH_2_-UiO-66 without apparent aggregation. The prepared Bi_2_S_3_@UiO-66-NH-S-Ag_44_ and MoS_2_@UiO-66-NH-S-Ag_44_ photocatalysts exhibited exceptional photocatalytic activity and recycling for solar degradation of MB.

## Experimental

### Chemicals

2-Aminoterephthalic acid (H_2_-BDC-NH_2_) linker, *N*,*N*-dimethylformamide (DMF), zirconium chloride (ZrCl_4_), concentrated HCl, and ethanol were purchased from Sigma–Aldrich. Bismuth nitrate pentahydrate (Bi(NO_3_)_3_·5H_2_O), sodium sulfide (Na_2_S·9H_2_O), ethylene glycol (EG), and carbamide were purchased from Sigma–Aldrich. Ammonium molybdate tetrahydrate (NH_4_)_6_Mo_7_O_24_·4H_2_O and thioacetamide (C_2_H_5_NS, 99%) were purchased from Alfa Aesar. 5,5′-Dithiobis(2-nitrobenzoic acid) (DTNBA, 99%), silver nitrate (AgNO_3_, 99%), sodium hydroxide (NaOH), sodium borohydride (NaBH_4_, 99.99% metals basis), and tetramethylammonium hydroxide (TMAH, 23%) were purchased from Sigma Aldrich. All chemicals were used without further purification. Methylene blue dye (MB) was used to determine the photocatalytic activity of the prepared photocatalysts.

### Synthesis of UiO-66-NH_2_

UiO-66-NH_2_ was synthesized as reported before by the Farha group^42^. Briefly, 10 mL conc. HCl was added dropwise over suspended ZrCl_4_ (1.25 g) in 50 mL DMF, and then the solution was sonicated for 20 min until fully dissolved. 2-Aminoterephthalic acid as linker (1.34 g) was dissolved in 100 mL DMF and added to the solution. The solution was sonicated for a further 20 min and then heated in an oven at 80 °C for 16–18 h. The resulting solid was filtered and washed with DMF (2 × 30 mL) and EtOH (2 × 30 mL). Finally, NH_2_-UiO-66 was dried in an oven at 70 °C overnight. The NH_2_-UiO-66 was used after activation at 150 °C for 12 h.

### Synthesis of Bi_2_S_3_ nanorods

Bi_2_S_3_ nanorods were synthesized according to a previously reported method^43^. In a typical process, two solutions were prepared, solution (A) consisted of 1.82 g Bi(NO_3_)_3_·5H_2_O that was added into 20 mL ethylene glycol (EG) under stirring for 30 min and 1.35 g Na_2_S was dissolved in 20 mL DI water to form solution (B). And then solution B was added dropwise into solution A, and a black suspension appeared during the process. After that, a carbamide solution (1.92 g CO(NH_2_)_2_ in 20 mL DI H_2_O) was added to the mixed solution as a pH modifier. The mixture was heated in a Teflon-lined stainless steel autoclave at 180 °C for 24 h. The product was washed with ethanol and DI water several times and dried at 60 °C to obtain Bi_2_S_3_ nanorods.

### Synthesis of MoS_2_ nanoparticles

Typically, 2.48 g of ammonium molybdate tetrahydrate (NH_4_)_6_Mo_7_O_24_·4H_2_O (2 mmol) and 1.2 g of thioacetamide C_2_H_5_NS (16 mmol) was dissolved in 75 mL DI water. The solution was stirred for one hour at room temperature, ultrasonicated for 10 min, and then heated in a Teflon-lined stainless steel autoclave at 180 °C for 24 h. The product was cooled and washed with DI water several times and dried at 80 °C to obtain MoS_2_ nanoparticles^44^.

### In-situ preparation of Bi_2_S_3_@UiO-66-NH_2_ and MoS_2_@UiO-66-NH_2_ heterojunctions

**Figure 1:**
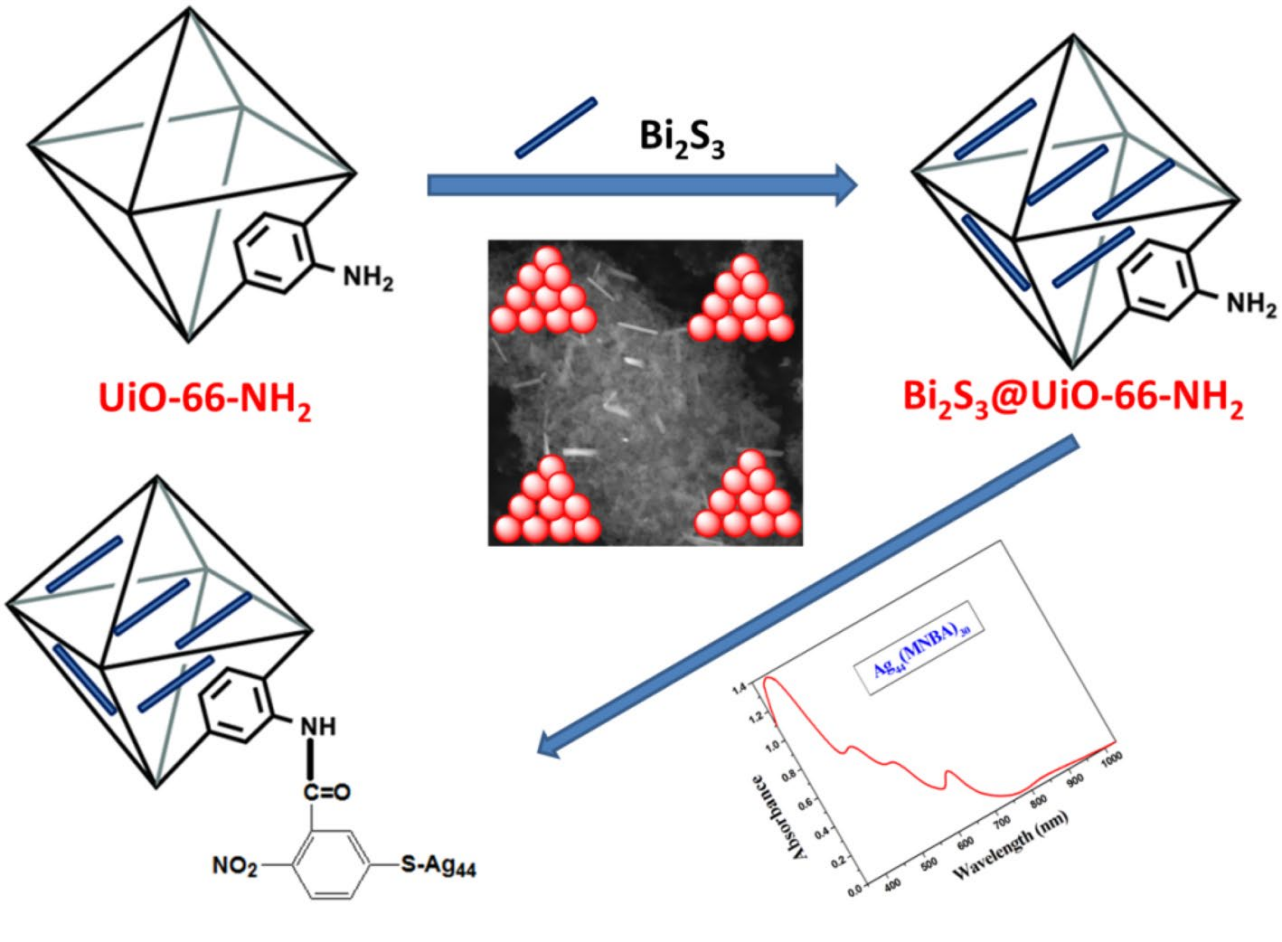
Scheme of the preparation of Bi_2_S_3_@UiO-66-NH-S-Ag_44_ photocatalysts.

The one-pot synthesis of the Bi_2_S_3_@NH_2_-UiO-66 and MoS_2_@NH_2_-UiO-66 is based on the same procedure used for the preparation of NH_2_-UiO-66 in section "Synthesis of UiO-66-NH_2_". The Bi_2_S_3_ and MoS_2_ in DMF were added to the NH_2_-UiO-66 precursors, to immobilize the Bi_2_S_3_ and MoS_2_ inside the NH_2_-UiO-66 frameworks. After that, the precipitate was filtered and washed with DMF (2 × 30 mL) and EtOH (2 × 30 mL). Finally, the Bi_2_S_3_@UiO-66-NH_2_ and MoS_2_@UiO-66-NH_2_ were dried in an oven at 70 °C overnight (Fig. 1). The loading percentage of the metal chalcogenides (Bi_2_S_3_ or MoS_2_) was around 3%.

### Synthesis and purification of Ag_44_(SC_6_H_4_O_4_N)_30_ nanoclusters (NCs)

Ag_44_(SC_6_H_4_O_4_N)_30_ was synthesized as reported before^45^. Briefly, 9.91 mg of 5,5′-dithiobis(2-nitrobenzoic acid) (DTNBA) was stirred in 20 mL NaOH aqueous solution (1 M). The disulfide bond was cleaved which was indicated by the formation of a dark yellow solution from 5-mercapto-2-nitrobenzoic acid (MNBA). 8.5 mg of AgNO_3_ (50 mmol) was dissolved in 5 mL DI water and added to the MNBA solution. The color was changed from dark yellow to greenish yellow indicating the formation of an Ag–S complex. A fresh NaBH_4_ solution (1 mg in 2 mL DI water) was then used to reduce the complex. The solution turned dark brown immediately and gradually changed to dark red under vigorous stirring for 4 h, indicating the formation of Ag_44_(SR)_30_ NCs. The clusters were purified by repeated precipitation with 50% methanol followed by repeated centrifugation at 9000 rpm for 10 min and decantation of the supernatant until it became colorless.

### Covalently anchoring of Ag_44_ on Bi_2_S_3_@UiO-66-NH_2_ and MoS_2_@UiO-66-NH_2_

100 mg Bi_2_S_3_@UiO-66-NH_2_ or MoS_2_@UiO-66-NH_2_ was dispersed in 40 mL DI water and sonicated for 30 min. 5 mg of Ag_44_(SC_6_H_4_O_4_N)_30_ nanoclusters in methanol was added over the suspension and sonicated for 30 min. The final solution was heated at 80 °C, for 2 h and then stirred overnight at room temperature. The products donated as Bi_2_S_3_@UiO-66-NH-S-Ag_44_ (Fig. 1) or MoS_2_@UiO-66-NH-S-Ag_44_ were collected by filtration and then dried in an oven overnight^17^.

### Photocatalytic studies of the prepared photocatalysts

The photocatalytic degradation of methylene blue (MB) solution using visible light was carried out to evaluate the photocatalytic activity of the prepared photocatalysts. A 450 W medium-pressure mercury lamp with a < 420 nm UV cut-off filter was used as a visible light source for the photocatalytic experiments, the lamp was fixed 10 cm away from the reaction system, as used in our previous work^5,6, 46–48^. 30 mg of the prepared photocatalysts were suspended in 50 mL of highly concentrated aqueous solution MB (50 ppm) under magnetic stirring. To establish an adsorption–desorption equilibrium, the reaction system was first kept in the dark for 60 min and then exposed to visible light for two hours. 5 mL aliquots from each sample were taken at the desired time intervals, followed by centrifugation and filtration to remove the photocatalyst. The decolorization of the MB solution was evaluated by measuring the change in its characteristic optical absorbance using an Evolution 300 UV–Vis spectrophotometer^5,6^. To check the advantages of the prepared photocatalysts and their applicability to reuse^5,6^, the photodegradation reaction of the MB solution was achieved with Bi_2_S_3_@UiO-66-NH-S-Ag_44_ photocatalyst. Then the photocatalyst was collected at the end of the reaction and reused for a second cycle and the process repeated so on till five cycles keeping all other parameters constant.

## Results and discussion

Atomically precise monodispersed thiol-protected silver nanoclusters [Ag_44_(MNBA)_30_] were synthesized using 5-mercapto-2-nitrobenzoic acid as a protecting ligand (Fig. S1). The used method produced monodisperse and stable silver nanoclusters in aqueous solution for at least 9 months at room temperature under ambient conditions. Electrospray ionization mass spectrometry (ESI–MS) was used to determine the composition, size, and monodispersity of the clusters^45^. The silver nanoclusters [Ag_44_(MNBA)_30_] showed at least five characteristic absorption peaks in the visible-NIR region with absorption maxima at 400, 480, 550, 650, and 850 nm (Fig. S2).

### Characterization of the prepared photocatalysts

The crystallinity phase composition, texture properties, structure morphology, and particle size of the prepared photocatalysts were characterized by X-ray diffraction (XRD), adsorption–desorption N_2_ isotherms, FE-SEM, and HR-TEM, respectively. The chemical structure and the stoichiometry and charge of the prepared photocatalysts were investigated by FT-IR and X-ray photoelectron spectroscopy (XPS), respectively. The UV–Vis diffuse reflectance spectroscopic analysis was used to investigate the optical absorption properties and the band-gap of the prepared photocatalysts^49^.

**Figure 2:**
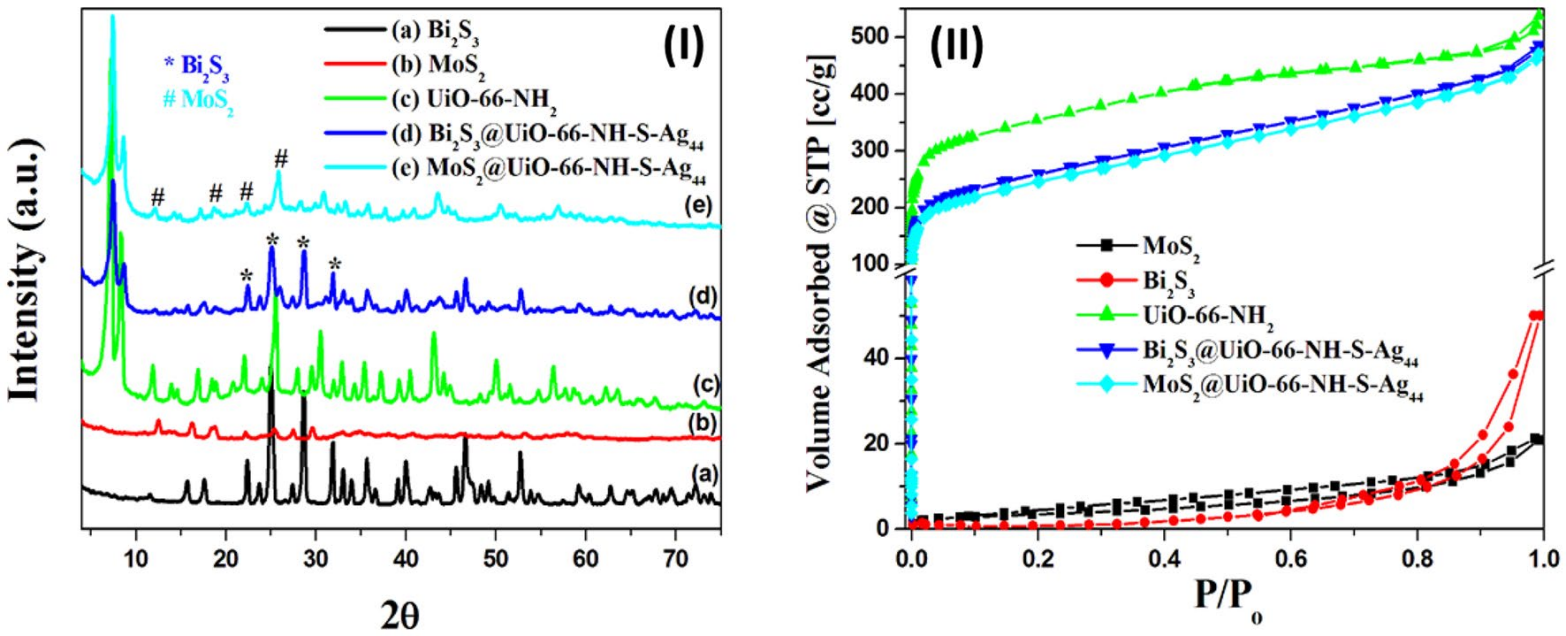
(**I**) X-ray diffractograms of the prepared photocatalysts, (**a**) Bi_2_S_3_, (**b**) MoS_2_, (**c**) UiO-66-NH_2_, (**d**) Bi_2_S_3_@UiO-66-NH-S-Ag_44_, and (**e**) MoS_2_@UiO-66-NH-S-Ag_44_. (**II**) The nitrogen adsorption–desorption isotherms of Bi_2_S_3_, MoS_2_, UiO-66-NH_2_, Bi_2_S_3_@UiO-66-NH-S-Ag_44_, and (**e**) MoS_2_@UiO-66-NH-S-Ag_44_.

The XRD patterns of the prepared photocatalysts were presented in Fig. 2I, which confirms the successful fabrication of Bi_2_S_3_ nanorods, MoS_2_ nanoparticles, NH_2_-UiO-66, MoS_2_@UiO-66-NH-S-Ag_44_, and Bi_2_S_3_@UiO-66-NH-S-Ag_44_ photocatalysts. The Bi_2_S_3_ nanorods exhibited XRD diffraction peaks at 2θ = 15.8°, 17.6°, 22.6°, 23.8°, 25.2°, 27.39°, 28.6°, 32°, 33.18°, 33.98°, 35.77°, 36.77°, 39.17°, 40.17°, 45.76° and 46.56°, corresponding to the (020), (120), (220), (101), (130), (021), (211), (221), (301), (311), (240), (231), (041), (141), (002) and (431) planes, respectively (Fig. 2Ia), which are matched well with the previous reported work^43^, and the JCPDS card no. 17-0320. The MoS_2_ shows weak intensity diffraction peaks in comparison to the diffraction peaks of Bi_2_S_3_, indicating the poor crystallinity and lower particle size of the prepared MoS_2_ nanoparticles (Fig. 2Ib), the XRD diffraction peaks of the MoS_2_ at 2θ = 12.6°, 16.35°, 19°, 25.4° and 29.7° are corresponding to the (002), (004), (100), (102) and (103) planes and the JCPDS card no. 65–0160^50^, respectively. The characteristic diffraction peaks at 2θ = 7.3°, 8.5°, and 25.7°^18,19^, corresponding to the (111), (200), and (531) planes of NH_2_-UiO-66, respectively (Fig. 2Ic). As shown in the Fig. 2Id the two components of the Bi_2_S_3_@UiO-66-NH-S-Ag_44_ photocatalyst were observed, which suggests the successful combination of the Bi_2_S_3_ nanorods and NH_2_-UiO-66. The diffraction peaks at 7.5° and 8.5° are identified for the NH_2_-UiO-66 and the diffraction peaks at 2θ = 22.6°, 25.2°, 28.6° and 32° (labeled with a ‘*’ mark) are corresponding to Bi_2_S_3_ (Fig. 2Id). The same case appeared in the MoS_2_@UiO-66-NH-S-Ag_44_, the characteristic diffraction peaks of the MoS_2_ are labeled with a ‘#’ mark (Fig. 2Ie). There is no characteristic XRD peak for silver nanoclusters (Ag_44_) in Fig. 2Id,e, due to its high dispersion inside the MOF's skeleton structure and the lower loading percentage^18^.

The textural properties of the prepared photocatalysts were investigated using the N_2_ adsorption–desorption isotherms at 77 K, as shown in Fig. 2II. The specific surface area (S_BET_) and the pore volume distribution of the prepared photocatalysts were determined using the Brunauer–Emmett–Teller (BET) equation and the Barrett- Joyner-Halenda (BJH) method^6,18^, respectively. The S_BET_ of the pure NH_2_-UiO-66 is 1106 m^2^/g with a total pore volume of 0.40 cm^3^/g^18^. The specific surface area and pore volume of the NH_2_-UiO-66 were decreased after loading with Bi_2_S_3_ or MoS_2_ and Ag_44_ as shown in Table 1. All of them showed isotherms belonging to type I with H4 hysteresis loops, corresponding to the IUPAC classification of the hysteresis loops, these materials have mesoporous pores^51^. However, the synthesized metal chalcogenides (Bi_2_S_3_ and MoS_2_) exhibited lower specific surface areas (Table 1). The surface areas of the prepared photocatalysts were measured by another method (T-method, S_t_). The values of S_t_ are equal S_BET_, which confirms the correct choice of the standard t-curves (Table 1).

**Table 1:** Surface area data for the prepared photocatalysts.

Catalysts	S_BET_ (m^2^ g^−1^)	S_t_ (m^2^ g^−1^)	Pore volume (cm^3^/g) × 10^–3^
NH_2_-UiO-66	1106	1106	400
Bi_2_S_3_@UiO-66-NH-S-Ag_44_	805	804	335
MoS_2_@UiO-66-NH-S-Ag_44_	769	770	312
Bi_2_S_3_	22	22	55
MoS_2_	8	8	20

**Figure 3:**
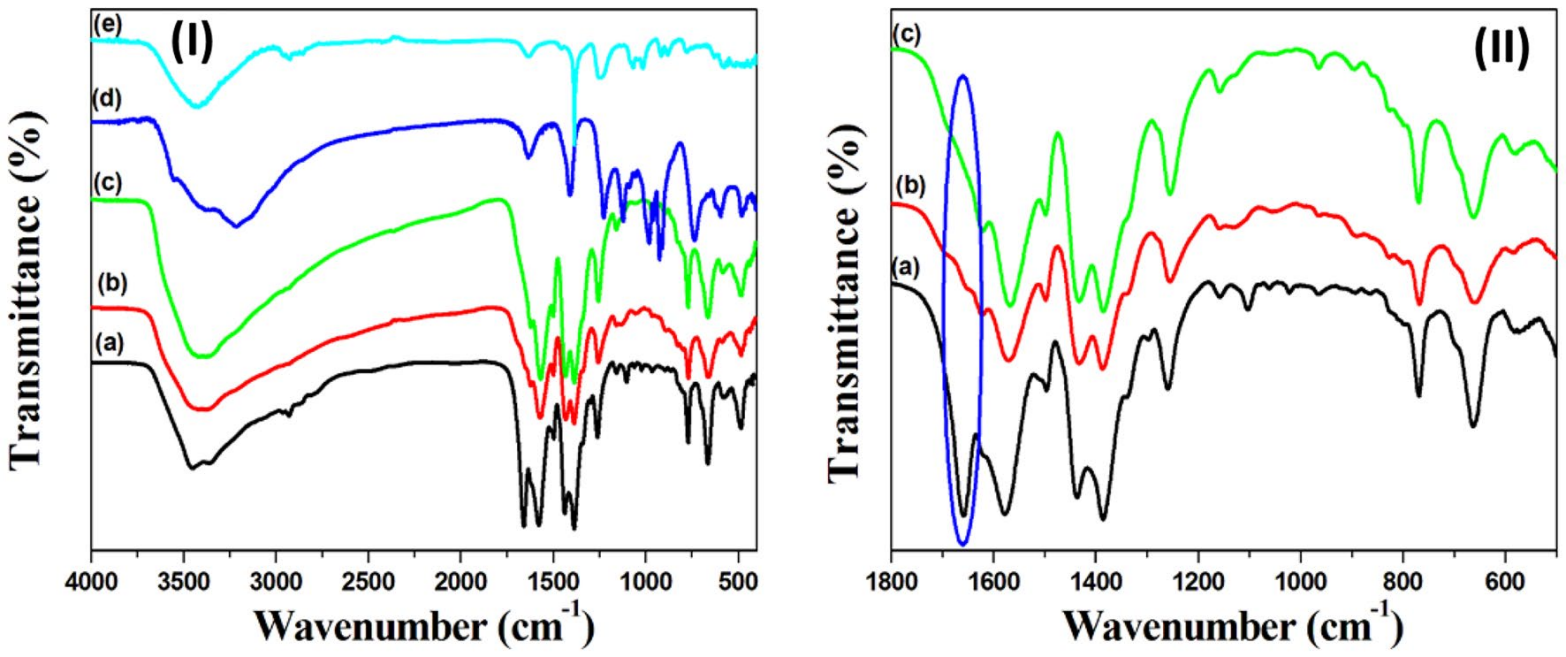
(**I**) FT-IR spectra of (**a**) UiO-66-NH_2_, (**b**) MoS_2_@UiO-66-NH-S-Ag_44_, (**c**) Bi_2_S_3_@UiO-66-NH-S-Ag_44_ (**d**) MoS_2_ and (**e**) Bi_2_S_3_. (**II**) FT-IR spectra of (**a**) UiO-66-NH_2_, (**b**) MoS_2_@UiO-66-NH-S-Ag_44_, (**c**) Bi_2_S_3_@UiO-66-NH-S-Ag_44_ from 500 to 1800 cm^−1^.

FTIR analysis was carried out to verify the formation of linkages between the NH_2_-UiO-66 and the protected silver nanoclusters [Ag_44_(MNBA)_30_] via a condensation reaction. Figure 3Ia shows the FTIR spectrum of the pristine NH_2_-UiO-66, where the absorption peaks at 3440 cm^−1^ and 1577 cm^−1^ are assigned to the amino N–H and carbonyl C=O groups, respectively^17^. The C–N stretching vibration modes show two absorption bands at around 1360 cm^−1^ and 1259 cm^−1^. The absorption bands between 768 cm^−1^ and 572 cm^−1^ are assigned to the Zr–O modes. The C = C skeletal vibration of the benzene ring shows an absorption peak at 1566 cm^−1^ in NH_2_-UiO-66^17^. No significant differences were observed between the FTIR spectra of MoS_2_@UiO-66-NH-S-Ag_44_ (Fig. 3Ib) and Bi_2_S_3_@UiO-66-NH-S-Ag_44_ (Fig. 3Ic) in comparison to NH_2_-UiO-66, confirming the existence of NH_2_-UiO-66 in the photocatalysts, instead of the absences of the N–H bending vibration peak of primary amines that observed in the region 1650–1580 cm^−1^ as shown in Fig. 3II, due to the covalent anchoring of the protected silver clusters throw the condensation reaction between the NH_2_ group of NH_2_-UiO-66 and the carboxylic group of the 5-mercapto-2-nitrobenzoic acid ligand.

**Figure 4:**
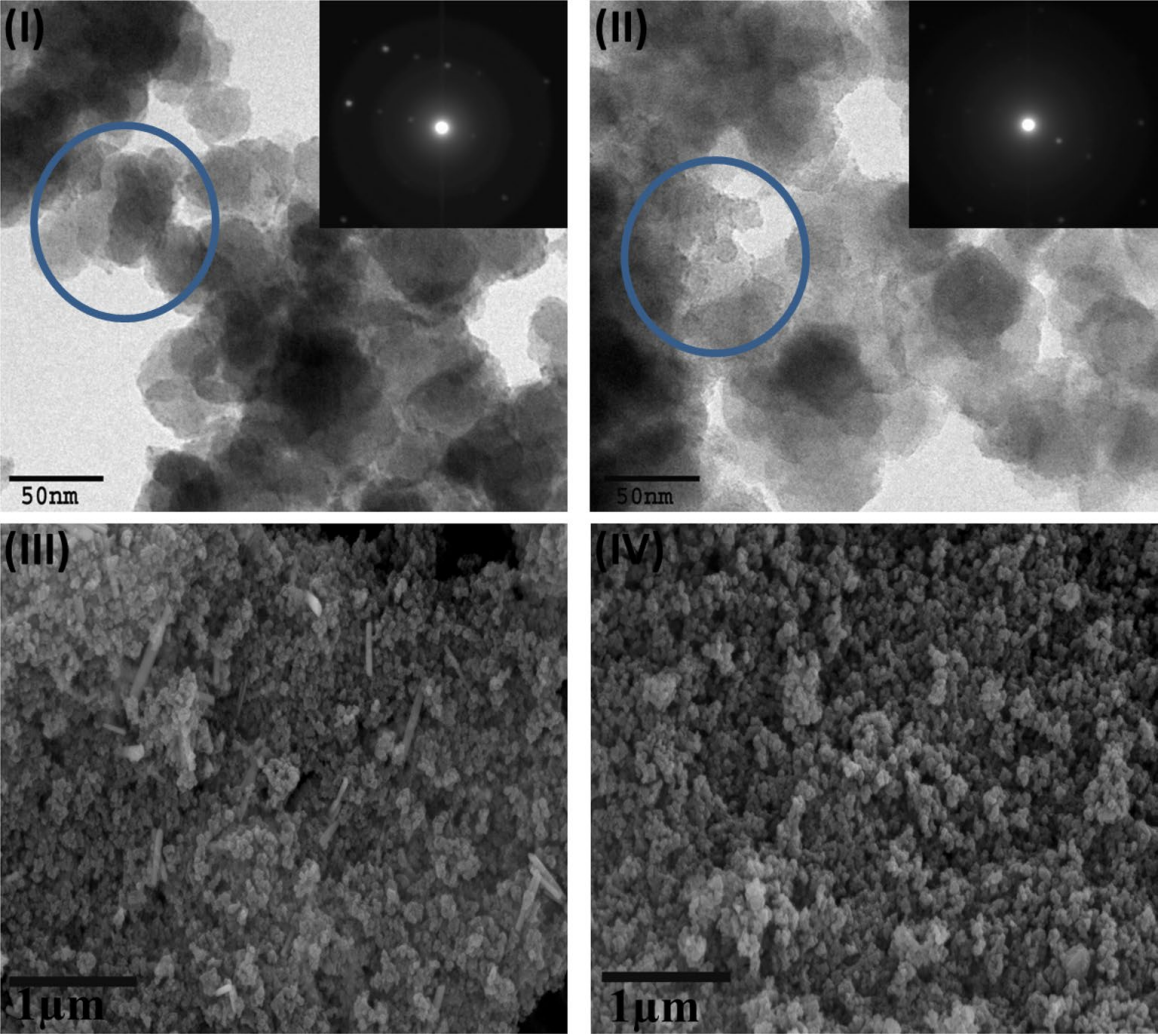
High resolution-transmission electron microscopy (HR-TEM) images of the Bi_2_S_3_@UiO-66-NH-S-Ag_44_ (**I**) and MoS_2_@UiO-66-NH-S-Ag_44_ (**II**). FE-SEM images of Bi_2_S_3_@UiO-66-NH-S-Ag_44_ (**III**) and MoS_2_@UiO-66-NH-S-Ag_44_ (**IV**).

The morphological characteristics of the Bi_2_S_3_@UiO-66-NH-S-Ag_44_ and MoS_2_@UiO-66-NH-S-Ag_44_ and the particle size of the loaded silver nanoclusters were identified by High resolution-transmission electron microscopy (HR-TEM) (Fig. 4I,II), respectively. The octahedral morphology with very smooth crystals of the pristine NH_2_-UiO-66, as reported before in our previous work^18,19^ does not appear in the case of Bi_2_S_3_@UiO-66-NH-S-Ag_44_ (Fig. 4I) and MoS_2_@UiO-66-NH-S-Ag_44_ (Fig. 4II), and the surface becomes rough, due to the complete encapsulation of the Bi_2_S_3_ and MoS_2_ by NH_2_-UiO-66. There are homogenous black dots inside the blue circle of the TEM images of Bi_2_S_3_@UiO-66-NH-S-Ag_44_ and MoS_2_@UiO-66-NH-S-Ag_44_, which refer to the silver nanoclusters (Ag_44_) with an average particle size of ~ 1.5 nm as shown in Fig. S1, Fig. 4I,II. The inset images in Fig. 4I,II refer to the crystallinity of the prepared Bi_2_S_3_@UiO-66-NH-S-Ag_44_ and MoS_2_@UiO-66-NH-S-Ag_44_ photocatalysts. The high magnification FE-SEM images of Bi_2_S_3_@UiO-66-NH-S-Ag_44_ (Fig. 4III) and MoS_2_@UiO-66-NH-S-Ag_44_ (Fig. 4IV) indicate that the Bi_2_S_3_ and MoS_2_ appear as nanorods and nanoparticles over the surface and inside the cavities of the NH_2_-UiO-66 without apparent aggregation.

**Figure 5:**
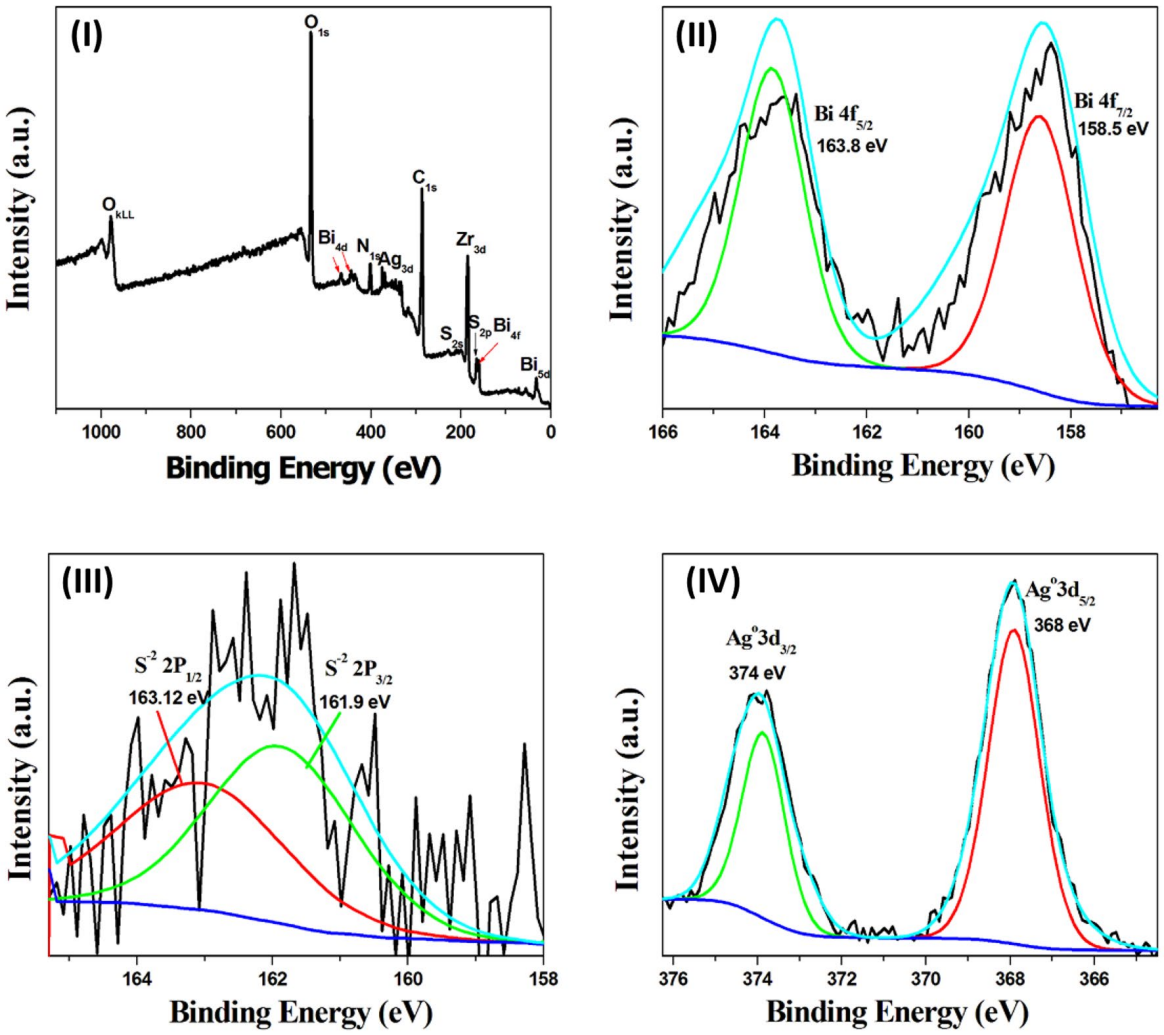
High-resolution X-ray photoelectron spectroscopy (HR-XPS) spectra of the Bi_2_S_3_@UiO-66-NH-S-Ag_44_ photocatalyst, (**I**) survey spectrum, (**II**) the XPS spectrum of Bi 4f., (**III**) the XPS spectrum of S 2p and (**IV**) the XPS spectrum of Ag 3d.

**Figure 6:**
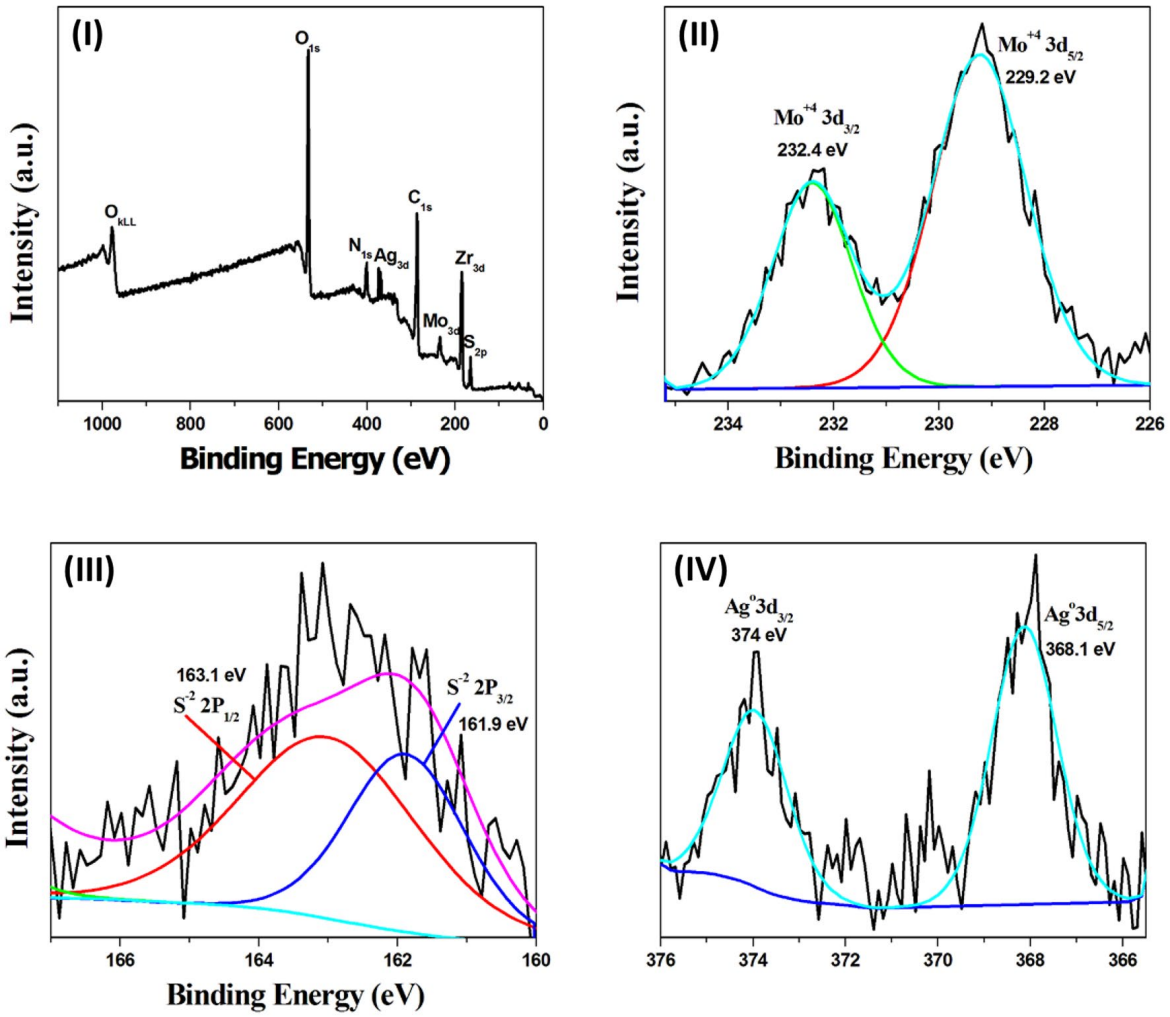
High-resolution X-ray photoelectron spectroscopy (HR-XPS) spectra of the MoS_2_@UiO-66-NH-S-Ag_44_ photocatalyst, (**I**) survey spectrum, (**II**) the XPS spectrum of Mo 3d, (**III**) the XPS spectrum of S 2p and (**IV**) the XPS spectrum of Ag 3d.

To estimate the valence state of the covalently anchoring silver nanoclusters and the elemental content of the prepared Bi_2_S_3_@UiO-66-NH-S-Ag_44_ (Fig. 5) and MoS_2_@UiO-66-NH-S-Ag_44_ (Fig. 6) photocatalysts, the X-ray photoelectron spectroscopy (XPS) measurements were conducted. Figure 5I displays a typical survey spectrum of the Bi_2_S_3_@UiO-66-NH-S-Ag_44_ and confirms the existence of Bi 4f, 4d and 5d, Zr 3d, S 2s and 2p, C 1s, O 1s, N 1s, and Ag 3d. The Zr, C, and O elements show the strongest peaks in the survey spectrum, with a small peak of N indicating the crystal lattice of NH_2_-UiO-66. To indicate the presence of the Bi_2_S_3_ in the prepared photocatalyst the high-resolution XPS spectra of Bi and S are analyzed separately, as shown in Fig. 5II,III, respectively. The binding energy peaks at 158.5 eV and 163.8 eV were ascribed for Bi 4f_7/2_ and Bi 4f_5/2_ (Fig. 5II) and the peaks at 161.9 eV and 163.12 eV observed for S 2p_3/2_ and S 2p_1/2_ transitions (Fig. 5III), respectively^1,17^. The chemical states of Bi and S were Bi^3+^ and S^2−^ in the loaded Bi_2_S_3_, which are following the previous literature^1,17^. The XPS analysis was used to determine the charge of the covalently anchoring Ag_44_ nanoclusters in the Bi_2_S_3_@UiO-66-NH_2_ photocatalyst. The XPS spectrum of Ag 3d shows two peaks at binding energy around 368 eV and 374 eV, corresponding to Ag 3d_5/2_ and Ag 3d_3/2_, respectively (Fig. 5IV). This is a characteristic peaks for the metallic silver (Ag^0^)^6^. This confirms the covalently anchoring silver nanoclusters (Ag_44_) have zero charge^45^.

The MoS_2_@UiO-66-NH-S-Ag_44_ photocatalyst shows the same XPS survey spectrum of the Bi_2_S_3_@UiO-66-NH-S-Ag_44_ with the same elements, instead of replacing the Bi with the Mo element (Fig. 6I). The high-resolution XPS spectrum of the Mo element exhibited two binding energy peaks at 229.2 eV and 232.4 eV that can be assigned to the Mo 3d_5/2_ and Mo 3d_3/2_^52^, respectively (Fig. 6II). The sulfur element in the MoS_2_@UiO-66-NH-S-Ag_44_ exhibited the same two binding energy peaks at 161.9 eV and 163.1 eV (Fig. 6III). Figure 6IV exhibited two binding energy peaks at 368.1 eV and 374 eV that are corresponding to metallic silver (Ag^0^).

**Figure 7:**
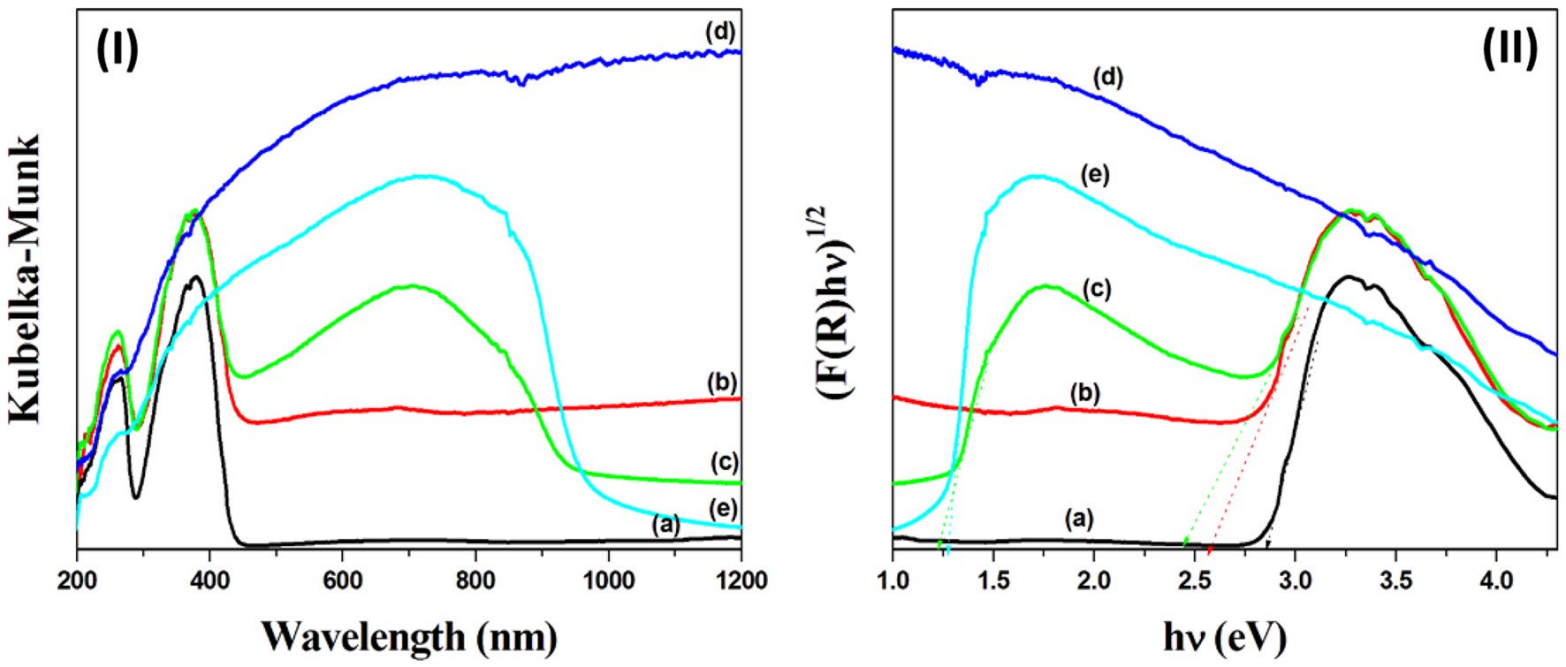
The UV–Vis-NIR diffuses reflectance spectra (**I**) and the plots of transformed K-M function **(II)** for the prepared photocatalysts (**a**) UiO-66-NH_2_, (**b**) MoS_2_@UiO-66-NH-S-Ag_44_, (**c**) Bi_2_S_3_@UiO-66-NH-S-Ag_44_ (**d**) MoS_2_ and (**e**) Bi_2_S_3_.

To measure the optical response of the pure UiO-66-NH_2_, Bi_2_S_3_, and MoS_2_ and the prepared photocatalysts (Bi_2_S_3_@UiO-66-NH-S-Ag_44_ and MoS_2_@UiO-66-NH-S-Ag_44_) the diffuse reflectance spectra (DRS) of the powder samples were recorded. The Kubelka–Munk (K-M) equation was used to correlate the absorbance of the samples with the diffuse reflectance^23^. Figure 7I exhibited the plot of the K-M function of the prepared photocatalysts depicting the K-M plots and Fig. 7II shows the corresponding (F(R)hν)^1/2^ vs. hν plot for the calculation of effective indirect band gap of the prepared photocatalysts.

Figure 7Ia exhibited the UV–Vis-NIR diffuse reflectance spectrum of the pure UiO-66-NH_2_ with two distinct peaks. The first peak centered at ~ 230 nm originated from the electron transition from the organic linker to the Zr–O cluster. The second strong peak centered at ~ 360 nm is attributed to the substitution of –NH_2_ in the organic linker^24^. The pure Bi_2_S_3_ shows a broad absorption peak that extended from UV to the near-infrared (NIR) region (300–1000 nm) and is centered at 705 nm (Fig. 7Ie), also the absorption range of the pure MoS_2_ covers the whole UV and visible light region (Fig. 7Id), which indicates the prepared metal chalcogenides have an excellent optical response. Compared to NH_2_-UiO-66, Bi_2_S_3_@UiO-66-NH-S-Ag_44_ (Fig. 7Ic) and MoS_2_@UiO-66-NH-S-Ag_44_ (Fig. 7Ib) exhibit an extended absorption in the whole visible light region and near-infrared (NIR) region, due to the special electronic distribution for the covalently anchoring silver nanoclusters (Ag_44_) and the doped optically active metal chalcogenides. The band gap values were calculated by the Kubelka–Munk from the extrapolation of the linear portion at (F(R)hν)^1/2^ = 0 providing the effective indirect band gap of the prepared photocatalysts (Fig. 7II). The band gaps of the pure NH_2_-UiO-66 and Bi_2_S_3_ were calculated to be 2.87 eV and 1.28 eV, respectively (Fig. 7II).

### Photocatalytic activity of the prepared photocatalysts under visible light irradiation

Methylene blue (MB) dye was chosen as a pollutant model to investigate the adsorption and photocatalytic activities of the prepared photocatalysts. A UV–Vis spectrophotometer (Evolution 300) was used to evaluate the photodegradation reaction. It is well known that the MB molecule is stable under visible light irradiation without photocatalyst^17^.

**Figure 8:**
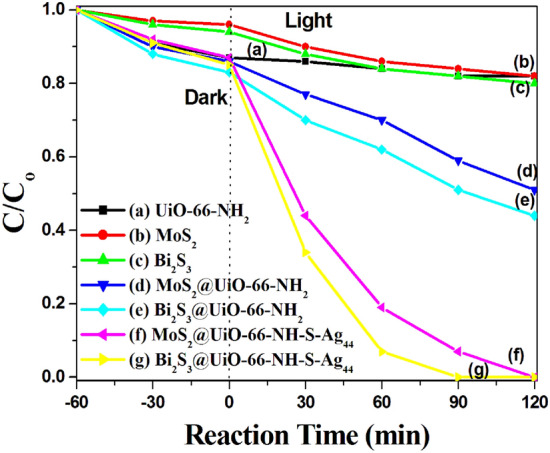
The photocatalytic decomposition of 50 ml (50 ppm) methylene blue (MB) dye over 30 mg of the prepared photocatalysts.

As shown in Fig. 8a, the NH_2_-UiO-66 sample shows the highest adsorption properties compared to the prepared MoS_2_ and Bi_2_S_3_ (Fig. 8b,c), respectively, due to its large surface area of 1106 m^2^/g and ordered nanosized channels, but the photodegradation activity of the NH_2_-UiO-66 is low due to its absorption properties is limited in the UV region, as confirmed by UV–Vis diffuse reflectance analysis (Fig. 7Ia).

The photocatalytic activity of the Bi_2_S_3_@UiO-66-NH_2_ and MoS_2_@UiO-66-NH_2_ heterojunctions is 56% and 49% in comparison to the pure UiO-66-NH_2_ (18%), indicating the synergistic effect between UiO-66-NH_2_ and Bi_2_S_3_ and MoS_2_ (Fig. 8e,d), respectively. Modification of UiO-66-NH_2_ with metal chalcogenides semiconductors (Bi_2_S_3_ and MoS_2_) plays an important role in the photocatalytic degradation of MB dye (Fig. 8e,d). Due to the Bi_2_S_3_ and MoS_2_ delay the recombination of the electron–hole pairs, enhance the conduction of charge carriers of UiO-66-NH_2_ and redshift the optical absorption of UiO-66-NH_2_ from the UV region (379 nm) to visible region at ~ 705 nm, as confirmed by the UV–Vis diffuse reflectance spectroscopic analysis (Fig. 7).

The MoS_2_@UiO-66-NH_2_ and Bi_2_S_3_@UiO-66-NH_2_ heterojunctions were covalently anchored with the size selected silver nanoclusters (Ag_44_) that show extremely photocatalytic activity in the photodegradation of the MB solution (Fig. 8f,g), respectively. The 50 ppm MB solution was destroyed over the MoS_2_@UiO-66-NH-S-Ag_44_ and Bi_2_S_3_@UiO-66-NH-S-Ag_44_ photocatalysts within 120 and 90 min stirring at room temperature under visible light illumination, respectively.

The extremely photocatalytic activity of the prepared photocatalysts was attributed to their amazing optical absorption properties. The Bi_2_S_3_@UiO-66-NH_2_ heterojunction absorbs in visible region at ~ 705 nm. The covalently anchoring zero charge silver clusters (Ag_44_) enhanced this absorption as shown in Fig. 7, due to the silver clusters have special optical properties, where it have five characteristic absorption peaks in the visible-NIR region with absorption maxima at 400, 480, 550, 650, and 850 nm, as shown in Fig. S2.

**Figure 9:**
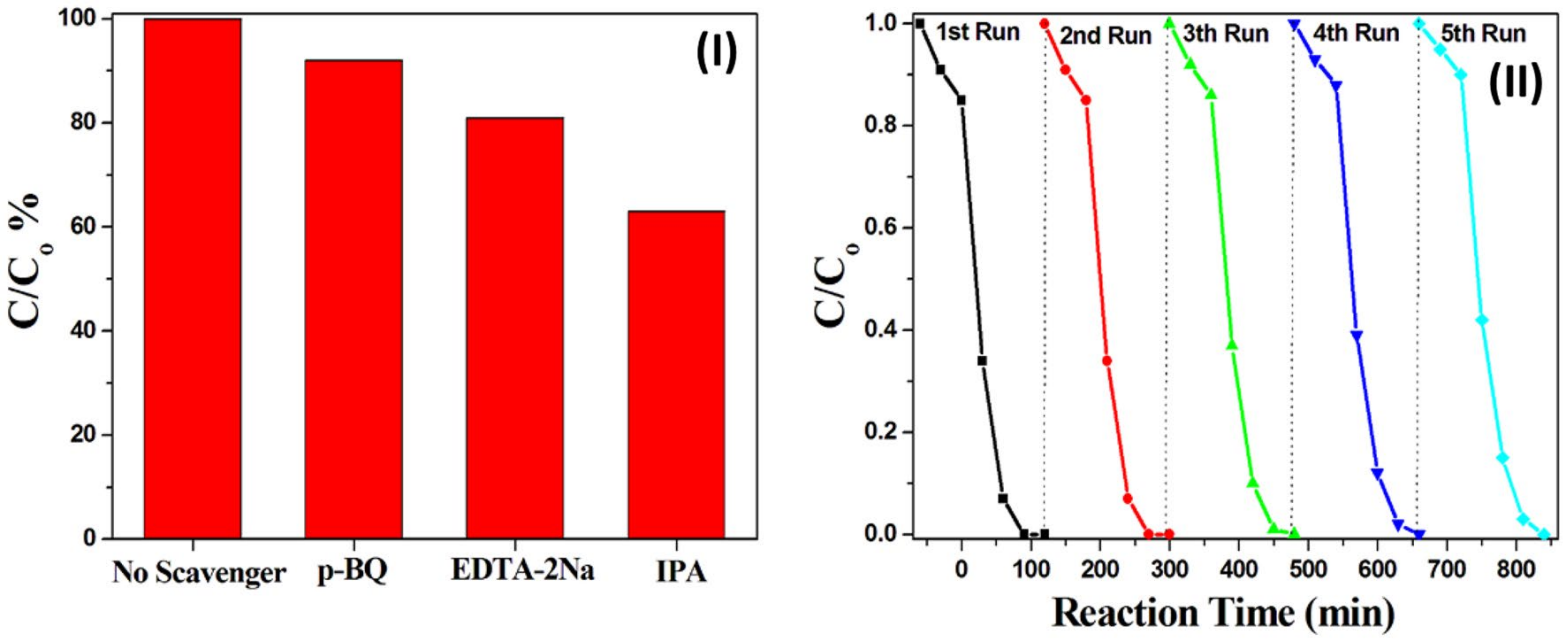
(**I**) Trapping experiments of active species over Bi_2_S_3_@UiO-66-NH-S-Ag_44_. (**II**) Recyclability effect of the Bi_2_S_3_@UiO-66-NH-S-Ag_44_ photocatalyst was studied for visible light degradation of MB solution.

The elemental trapping experiments have been performed to investigate the contribution of active species during the photocatalytic degradation of MB. P-benzoquinone (BQ), ethylene diamine tetraacetic acid disodium (EDTA-2Na) and isopropanol (IPA) were used as scavengers to quench the superoxide radical O_2_^**·**−^, photogenerated holes h^+^ and free radical hydroxide **·**OH, respectively. As shown in Fig. 9I, the photodegradation of MB over Bi_2_S_3_@UiO-66-NH-S-Ag_44_ photocatalyst without any scavenger reached 100% under visible irradiation for 90 min. The addition of IPA decreased the activity to 63%, while the degradation efficiency quenched to 92% and 81% when using p-BQ and EDTA-2Na, respectively. These results indicate that ⋅OH radical is the major active species for the MB degradation reaction. However, h^+^ and O_2_^**·**−^ have a minor effect on the photocatalytic process^8^.

The covalently anchoring silver nanoclusters on the MoS_2_@UiO-66-NH_2_ and Bi_2_S_3_@UiO-66-NH_2_ heterojunctions are not only enhancing the photocatalytic activity but also the recyclability properties. Where, Bi_2_S_3_@UiO-66-NH-S-Ag_44_ photocatalyst shows a very good activity for at least five catalytic runs without any loss in the photocatalytic degradation of MB (Fig. 9II). This means, these photocatalysts possess high stability and may be reusable for at least 5 runs, showing a good potential for industrial applications. This recyclability efficiency confirms a very low silver clusters leaching from the MoS_2_@UiO-66-NH_2_ and Bi_2_S_3_@UiO-66-NH_2_ surface. That was confirmed by measuring the XPS analysis for the catalyst before and after each recycling run, where the percentage of silver clusters remains nearly constant.

In the light of the previous discussion, the possible mechanism for MB photodegradation over the Bi_2_S_3_@UiO-66-NH-S-Ag_44_ photocatalyst is shown in Fig. S3. Under visible light illumination, UiO-66-NH_2_ modified by Bi_2_S_3_ and Ag_44_ was excited, leading to the generation of charge carriers. The photoexcited electrons on the conduction band (CB) of Bi_2_S_3_ could transfer directly to the CB of the UiO-66-NH_2_. Meanwhile, the valence band (VB) of UiO-66-NH_2_ (2.27)^53^ is more positive than Bi_2_S_3_ (1.48)^54^, thus the photoexcited holes migrate in the reverse direction of electrons (Fig. S3).

A comparison among different metal chalcogenides and Metal–organic frameworks photocatalysts that were reported as photocatalysts for the photodegradation of organic dyes is tabulated in Table 2.

**Table 2:** Comparison table on photodegradation of MB dye.

Photocatalysts	Pollutant content (mg/L)	Light Source	% of Degradation	Refs
Ca/TiO_2_/NH_2_-MIL-125	40	300 W Xe lamp	86.2	^55^
CoS_x_/NH_2_-MIL-125	20	300 W Xe lamp	95.4	^56^
BiOBr/NH_2_-MIL-125	20	500 W Xe lamp	93	^57^
MoS_2_/TiO_2_	5	400 W Xe Lamp	99.3	^58^
CuxS/TiO_2_	10	Visible light	95	^59^
CdS/TiO_2_	12	160 W Hg lamp	93.8	^60^
PbS/GR/TiO_2_	1 × 10^–4^ M	Visible light	41	^61^
MoS_2_@UiO-66-NH-S-Ag_44_	50	450 W Hg lamp Visible light	99.8	This work
Bi_2_S_3_@UiO-66-NH-S-Ag_44_	50	450 W Hg lamp Visible light	100	This work

## Conclusion

In summary, we have successfully prepared Bi_2_S_3_@UiO-66-NH-S-Ag_44_ and MoS_2_@UiO-66-NH-S-Ag_44_ photocatalysts through a condensation reaction for solar degradation of MB dye. Where the size selected silver nanoclusters [Ag_44_(MNBA)_30_] are anchored onto the Bi_2_S_3_@UiO-66-NH_2_ and MoS_2_@UiO-66-NH_2_ surface through a covalent interfacial reaction between the amine groups of UiO-66-NH_2_ and the carboxylic groups of the 5-mercapto-2-nitrobenzoic acid ligand. The covalently anchoring silver nanoclusters on the UiO-66-NH_2_ surface were confirmed by FTIR analysis, where the N–H bending vibration peak at 1650–1580 cm^−1^ was disappeared in the MoS_2_@UiO-66-NH-S-Ag_44_ and Bi_2_S_3_@UiO-66-NH-S-Ag_44_ FTIR spectra. The prepared Bi_2_S_3_@UiO-66-NH-S-Ag_44_ and MoS_2_@UiO-66-NH-S-Ag_44_ photocatalysts exhibited amazing photocatalytic activity and recyclability in the photodegradation of MB dye. The super photocatalytic performance of the prepared photocatalysts was attributed to the interfacial compactness of the silver nanoclusters in the heterojunction structure, which does not suffer from any leaching. Moreover, the doping of UiO-66-NH_2_ with Bi_2_S_3_ and silver clusters shift the optical absorption properties of UiO-66-NH_2_ from the UV region (379 nm) to the visible region at ~ 705 nm, as confirmed by the UV–Vis-NIR diffuse reflectance spectroscopic analysis. The prepared photocatalysts have high crystallinity properties as confirmed by XRD analysis and their textural properties were investigated with nitrogen adsorption–desorption isotherms at − 196 °C.

### Supplementary Information

Supplementary Information.

## Data Availability

All data generated or analyzed during this study are included in this published article (and its supplementary information files).
