# Supplementary material for: Covalently anchoring silver nanoclusters Ag44 on modified UiO-66-NH2 with Bi2S3 nanorods and MoS2 nanoparticles for exceptional solar wastewater treatment activity
Source: Sci Rep. 2023 Oct 17;13:17634. doi: 10.1038/s41598-023-44819-8 (PMC10582164; doi:10.1038/s41598-023-44819-8)
Supplement: Supplementary file 1 — Supplementary Information. [file 41598_2023_44819_MOESM1_ESM.docx]

**Supplementary Information**

**Covalently anchoring silver nanoclusters Ag_44_ on modified UiO-66-NH_2_ with Bi_2_S_3_ nanorods and MoS_2_ nanoparticles for exceptional solar wastewater treatment activity**

**Mostafa Farrag***

Chemistry Department, Faculty of Science, Assiut University, 71515 Assiut, Egypt

*[mostafafarrag@aun.edu.eg](mailto:mostafafarrag@aun.edu.eg)

**Instrumentation and characterization**

Adsorption–desorption isotherms of liquid nitrogen at −196 ^o^C are obtained using a Quantachrome (Nova 3200 series) multi-gas adsorption apparatus. Prior to analysis, the samples were outgassed at 150 ^o^C for 3 hrs. Specific surface areas are calculated from these isotherms by applying the BET equation. S_t_ Values are calculated using V_a–t_ plots of de Bore. Powder X-ray diffraction (XRD) is performed on a Philips X-ray powder diffractometer, model pw 2013/00. Ni-filtered Cu Kα with a wavelength of λ = 1.541838 Å was used as a constant source of radiation. The generator was operated at 35 kV and 20 mA, and the diffractometer at 50 diverting and receiving slits and a scan rate of 20 mm/min. Fine powder samples were loaded on a quartz plate holder by spreading the powders as a smooth thin layer on the plate. For all diffractograms, the following settings were used: scan range 2–70^o^ (2θ), scan step 0.06^◦^. The surface electronic states are investigated by using XPS K-ALPHA (Thermo Fisher Scientific, USA) with monochromatic X-ray Al K-alpha radiation -10 to 1350 e.v spot size 400 micro m at pressure 10^-9^ mbar with full-spectrum pass energy 200 e.v and narrow-spectrum 50 e.v. The XPS data are calibrated internally by fixing the binding energy (BE) of the C1s peak at 284.6 eV. For TEM measurements, solutions with a concentration of 1−2 mg/mL are prepared by dissolving the samples in DI water. A droplet of these sample solutions is cast onto carbon-coated copper grids. The solvent is then allowed to evaporate slowly. HR-TEM images are obtained with a JEOL JEM 2010 with a LaB6-Cathode electron microscope operating at an acceleration voltage of 200 kV. The images are then analyzed by using Image J software (version 1.44). Filed Emission-Scanning electron microscopy (FE-SEM, QUANTA FEG 250 - Made in the Netherlands) was used for the investigation and examination of the topographical features of the prepared photocatalyst. The UV–Vis-NIR diffuse reflectance spectroscopic (JASCO, ISN-470) analysis was used to investigate the optical absorption properties and the band gaps of the prepared photocatalysts. A 450 W medium-pressure mercury lamp with a *<*420 nm UV cut-off filter (visible light) was used as a light source for the photocatalytic experiments, the lamp was fixed at 10 cm away from the reaction system. The photodegradation rate of the MB solution was followed with a double-beam spectrophotometer (Evolution 300) at ambient temperature from 350 to 900 nm.


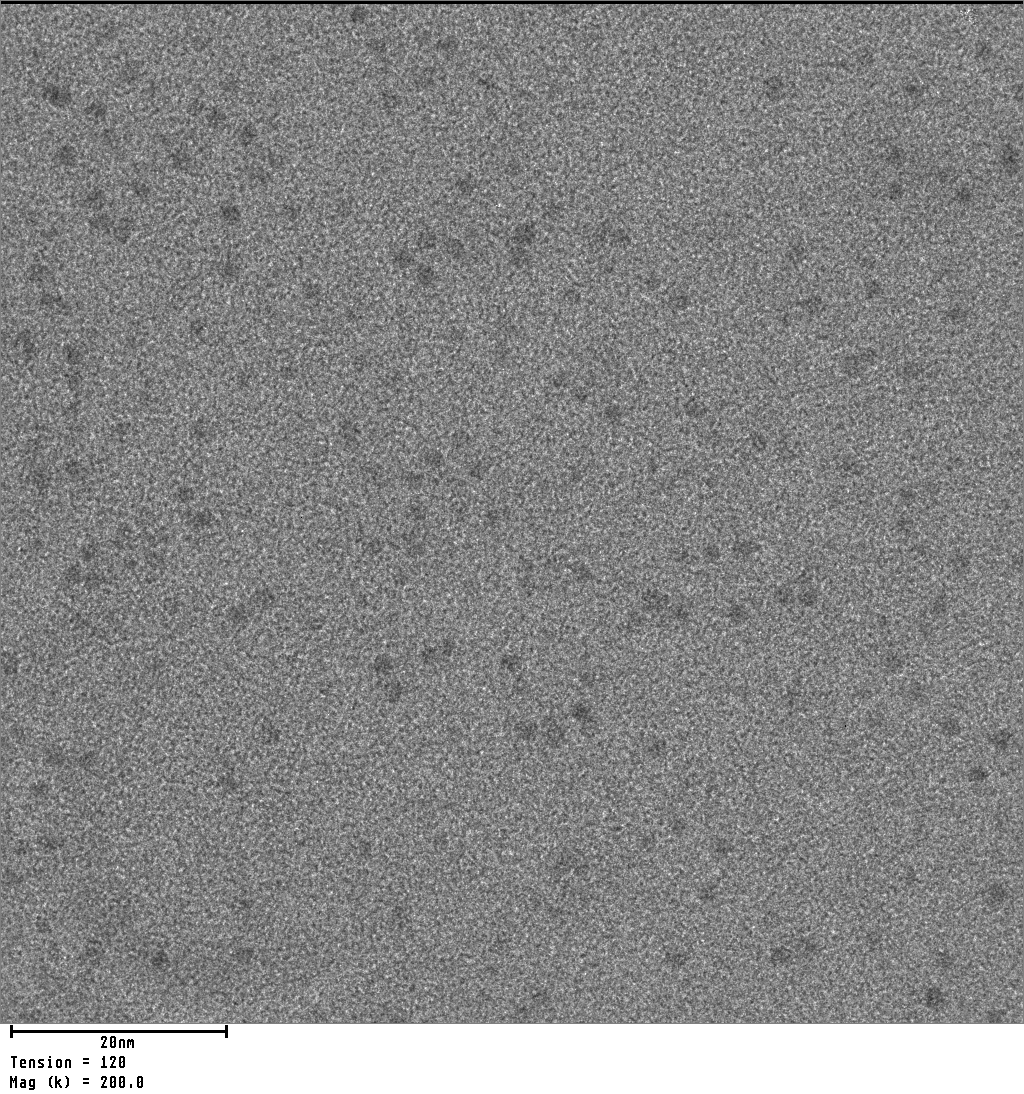


Fig. S1: HR-TEM of the Ag_44_(MNBA)_30_ nanoclusters.





**Fig. S2**. The UV-Vis spectrum of the Ag_44_(MNBA)_30_ nanoclusters.


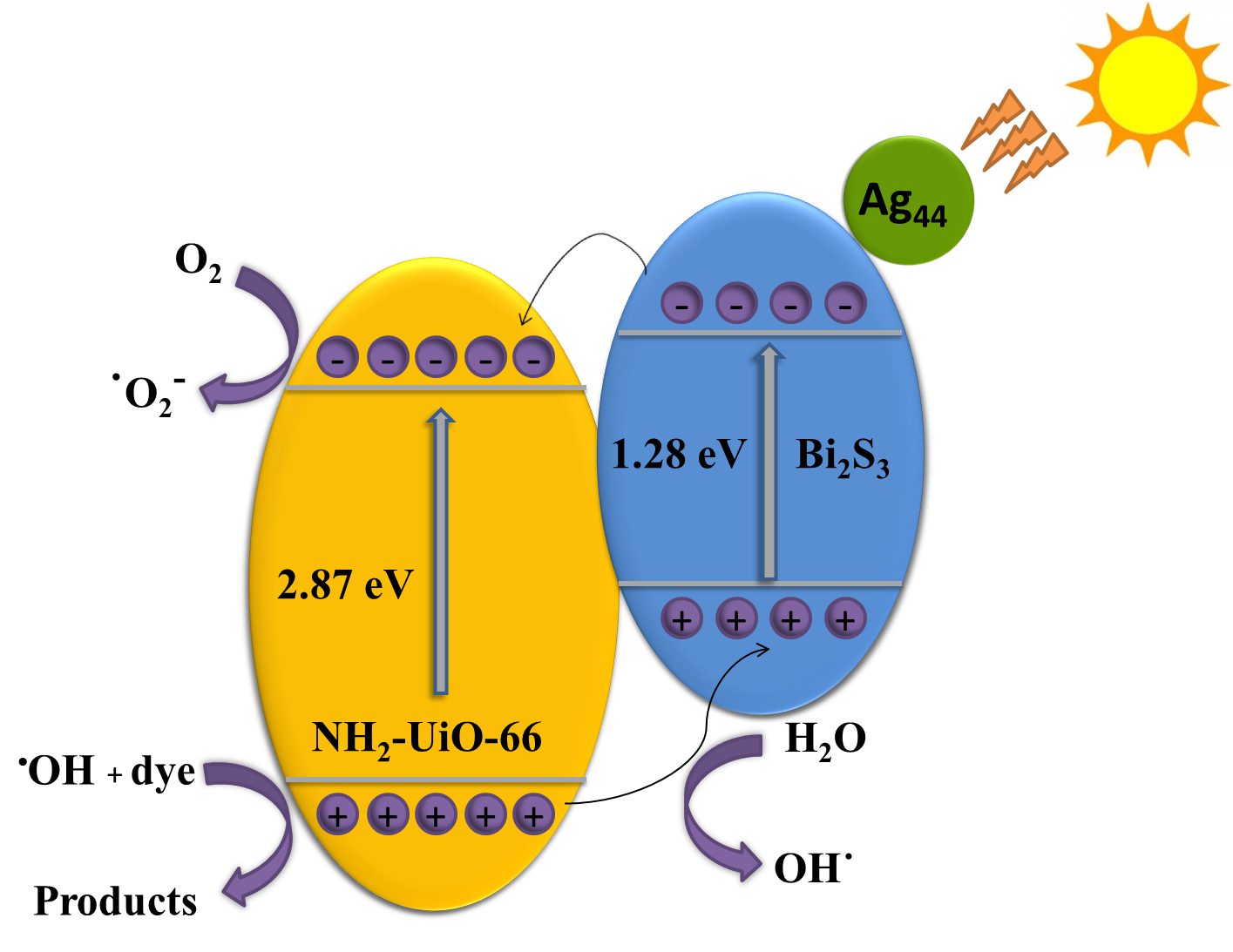


**Fig. S3: Mechanism of photocatalytic degradation of MB over Bi_2_S_3_@UiO-66-NH-S-Ag_44_ photocatalyst.**
